# Evaluation criteria for the assessment of occupational diseases of the lumbar spine - how reliable are they? -

**DOI:** 10.1186/s12891-019-2878-4

**Published:** 2019-10-27

**Authors:** Benjamin Ulmar, Julia Wölfle-Roos, Tugrul Kocak, Alexander Brunner

**Affiliations:** 1 BG Trauma Center Tübingen, Schnarrenbergstrasse 95, 72076 Tübingen, Germany; 2 Department of Orthopaedics, University of Ulm, Oberer Eselsberg 45, 89081 Ulm, Germany; 3 Department of Orthopaedics, Medical University Innsbruck, Anichstrasse 35, 6020 Innsbruck, Austria

**Keywords:** Occupational disease of the lumbar spine, Intervertebral disc height on plain x-rays, Hurxthal, Intervertebral osteochondrosis, Vertebral osteosclerosis, Worker compensation

## Abstract

**Background:**

In 2005, the German Association of Occupational Accident Insurance Funds (DGUV) defined radiological evaluation criteria for the assessment of degenerative occupational diseases of the lumbar spine. These include the measurement of intervertebral osteochondrosis and classification of vertebral osteosclerosis, antero-lateral and posterior spondylosis, and spondyloarthritis via plain radiography. The measures currently remain in daily use for determining worker compensation among those with occupational diseases. Here, we aimed to evaluate the inter- and intra-observer reliability of these evaluation criteria.

**Methods:**

We enrolled 100 patients with occupational degenerative diseases of the lumbar spine. Native antero-posterior and lateral radiographs of these patients were evaluated according to DGUV recommendations by 4 observers with different levels of clinical training. Evaluations were again conducted after 2 months to assess the intra-observer reliability.

**Results:**

The measurement of intervertebral osteochondrosis showed good inter-observer reliability (ICC: 0.755) and excellent intra-observer reliability (ICC: 0.827). The classification of vertebral osteosclerosis exhibited moderate kappa values for inter-observer reliability (*К*: 0.426) and intra-observer reliability (*К*: 0.441); the remaining 3 criteria showed poor inter- and intra-observer reliabilities.

**Conclusion:**

The measurement of intervertebral osteochondrosis and classification of vertebral osteosclerosis showed adequate inter- and intra-observer reliability in the assessment of occupational diseases of the lumbar spine, whereas the classification of antero-lateral and posterior spondylosis and spondyloarthritis stage exhibited insufficient reliability. Hence, we recommend the revision of the DGUV recommendations for the evaluation of occupational diseases of the lumbar spine.

## Background

Low back pain is one of the most common health problems worldwide [1, 2], but leads to a particularly significant healthcare burden in industrial countries [2, 3]. In Germany, 26% of all individuals participating in the national health insurance system require medical treatment for back pain at least once a year [4]. In addition to anatomical abnormalities and psychological disorders, occupational activity has been recognized as a major risk factor for the development of chronic low back pain [5–8]. Overall, approximately 20% of all occupational activities appear to be associated with pathological spinal load, which could lead to early degenerative lumbar disc disease followed by chronic pain [9]. In Germany, the degree of load-related degeneration of the lumbar spine is relevant in the determination of worker compensation. Therefore, in 1993, the Federation of Commercial Occupational Insurance Associations (HVBG)—subsequently renamed as the German Association of Occupational Accident Insurance Funds (DGUV)—defined occupational disease no. 2108 that describes the development of deep lumbar back pain as a result of degeneration of the intervertebral discs due to non-physiological occupational load [10]. However, there are very few objective and reliable parameters for assessing the severity of occupational lumbar spinal diseases.

To overcome this problem, in 2000, 2 projects were initiated to improve our scientific knowledge regarding the evaluation of occupational spinal diseases. First, the German Spine Study evaluated the epidemiological dose-effect-relationship between occupational working exposure and the development of degenerative spinal diseases related to the intervertebral disc [11]. Based on these data, the “Mainz-Dortmund-Dose-Model” was developed as a uniform algorithm to correlate work load and degenerative changes of the lower back [12–14]; this model is still currently used. Second, an interdisciplinary working group developed consensus recommendations regarding the following topics [10]: clinical und morphological parameters that correlate with load-related symptoms; causal factors related to the development of occupational spinal diseases; criteria for evaluating whether a patient needs to discontinue the stressful occupational activity; and criteria for assessing a reduction in earning capacity The recommendations of this working group were published in 2005, and remain in use at present [10]. The researchers defined 5 radiologic parameters for the assessment of pathologies of the intervertebral disc, evaluated using plain anteroposterior (AP) and lateral lumbar spinal radiographs, including intervertebral osteochondrosis, vertebral osteosclerosis, anterolateral and posterior spondylosis, and spondyloarthritis.

In Germany, orthopedic and trauma surgeons regularly examine patients with work-related pain and offer expert opinions to the DGUV on the severity of work-related degenerative spinal diseases. Thus, the spinal radiographs of thousands of patients are evaluated annually for the presence of intervertebral osteochondrosis, vertebral osteosclerosis, anterolateral and posterior spondylosis, and spondyloarthritis, to assess for the presence of occupation-related pathologies of the lumbar intervertebral discs. Nevertheless, despite their frequent use, the reliability of these parameters has not yet been evaluated. In the present study, we aimed to evaluate the inter- and intra-observer reliability of the radiological parameters, including intervertebral osteochondrosis, vertebral osteosclerosis, anterolateral and posterior spondylosis, and spondyloarthritis, using plain AP and lateral standing radiographs of the lumbar spine.

## Methods

### Study patients

Study patients were retrospectively identified from the spinal database of the Department of Orthopaedics of the University of Ulm. All patients who had been treated between June 1, 2015, and December 31, 2015, for the diagnosis of “occupational intervertebral disc disease” were evaluated. The inclusion criteria for the study included a patient’s age less than 65 years and the availability of native AP and lateral radiographs of the lumbar spine in the standing position within the last year.

Patients with distinct scoliosis (Cobb angle > 10°), marked spondylolisthesis (more than Meyerding Grade II), osteoporosis (t-score < 2), history of vertebral fractures, previous spinal surgery, or spinal tumors were excluded. Moreover, patients with severe deformities of the vertebral bodies or systemic endocrinological diseases that could potentially affect bone metabolism were excluded. According to these criteria, a total of 26 patients (18 men and 8 women) were excluded from the study.

Finally, 100 patients (63 men and 37 women) with a mean age of 55 years (range, 44–65 years) were included in the evaluation.

### Radiological evaluation criteria for occupational diseases of the lumbar spine related to the intervertebral discs

#### Intervertebral osteochondrosis

**Fig. 1:**
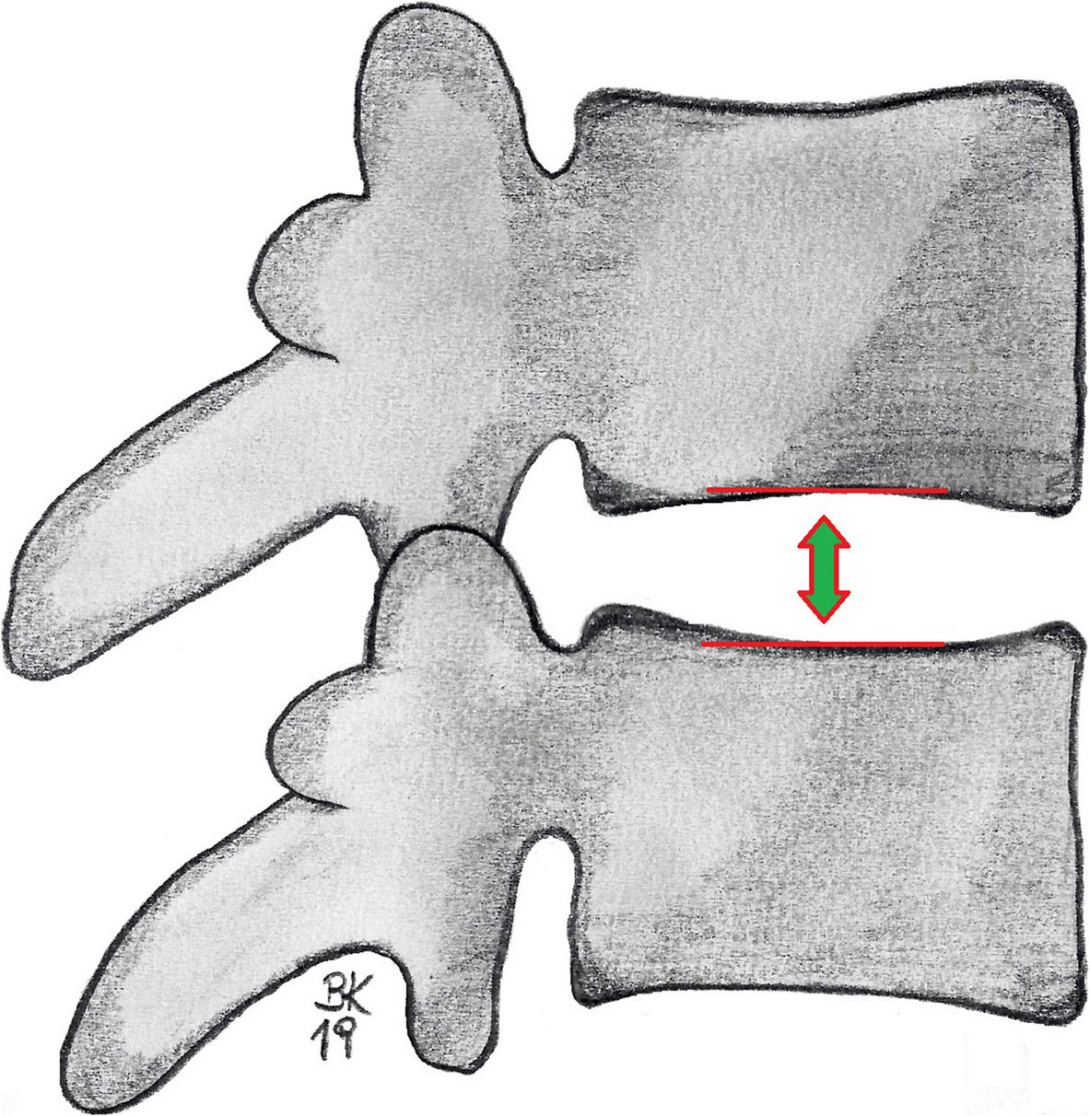
Evaluation of the intervertebral disc height for orthogonal projected lumbar segments according to Hurxthal

Intervertebral osteochondrosis describes the degenerative decrease of the height of the intervertebral disc. In general, the following relationships between the heights of the intervertebral lumbar disc spaces have been observed: L1/2 < L2/L3 < L3/L4 < L4/L5 > L5/S1 [15]. The German Association of Occupational Accident Insurance Funds recommends the method proposed by Hurxthal as the standard technique for measuring intervertebral disc space in patients with occupational diseases [10, 16]. Therefore, in orthogonally projected segments, the intervertebral disc space is measured in the axial direction as the maximum distance between the superior and inferior endplates of the opposite vertebral bodies (Fig. 1).

**Fig. 2:**
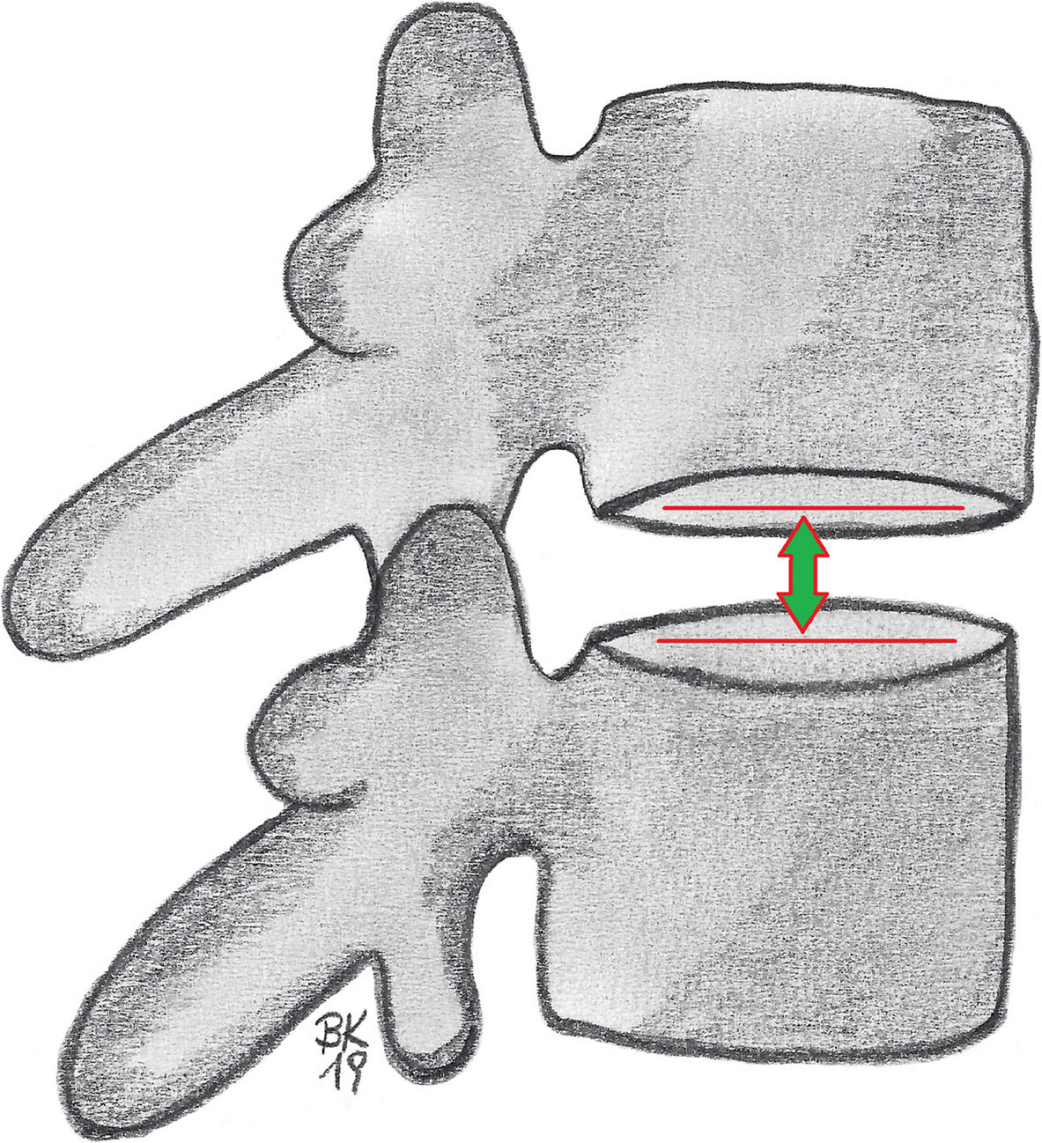
Evaluation of the intervertebral disc height for non-orthogonal projected lumbar segments according to Hurxthal

If the segment is not projected orthogonally, the disc space is measured between the anterior-posterior midlines of the projected oval planes of the superior and inferior endplates (Fig. 2).

#### Vertebral osteosclerosis

Vertebral osteosclerosis describes an abnormal hardening of the bone and an elevation of bone density in the vertebral endplates, which may extend into the cancellous bone of the vertebral body. For the assessment of osteochondrosis, 2 stages are considered based on the appearance on radiographs [10]: Stage 1, visible increase in the bone density of endplates without extension into cancellous bone by 2 mm; and Stage 2, extension of the osteosclerosis by > 2 mm into the cancellous bone of the vertebral body.

#### Spondylosis

Spondylosis describes a degenerative process associated with the development of spinal osteophytes. For the assessment of anterior and lateral spondylosis, 4 stages are considered based on the extension of osteophytes [10]: Stage 1, extension of osteophytes < 2 mm; Stage 2, extension of osteophytes > 2–5 mm; Stage 3, extension > 5 mm with partial bridging of the intervertebral disc space; and Stage 4, complete bridging of the intervertebral disc space.

For the assessment of posterior spondylosis, 2 stages were considered based on the extension of osteophytes: Stage 1, extension of ostophytes < 2 mm; and Stage 2, extension of osteophytes > 2 mm.

#### Spondyloarthritis

Spondyloarthritis is defined as osteoarthritis of the small intervertebral joints. Two stages of spondyloarthritis were considered: Stage 1, visible osteosclerosis of the facet joints; and Stage 2, osteosclerosis of the facet joints with additional hypertrophy of the articular process.

### Radiological evaluation

The patients’ AP and lateral lumbar spine radiographs were uploaded into a Picture Archiving and Communication System (Centricity PACS IW™, General Electric Health Care, Leeds, England). All identification marks were removed by an individual not part of the study team. To consider any possible effect of the observers’ experience on the assessment of spine radiographs we chose four observers with different levels of clinical training, including an orthopedic surgery resident (W), a consultant for orthopedic surgery (K), a consultant for trauma surgery (B), and a fellowship-trained spinal surgeon with several years of experience in the treatment of degenerative spinal diseases (U). The observers evaluated the radiographs independently and in random order. They measured the intervertebral disc spaces as proposed by Hurxthal, and classified the stages of vertebral osteosclerosis, anterolateral and posterior spondylosis, and spondyloarthritis [10, 16]. The assessment was conducted again after 2 months using the radiographs in random order.

### Statistical analysis

Inter- and intra-observer reliabilities were assessed by calculating the intra-class correlation coefficients (ICC) for linear data (intervertebral disc space) and the kappa coefficients (*К*) as proposed by Cohen for nominal data (vertebral osteosclerosis, spondylosis, and spondyloarthritis) [17]. Interpretation of the ICC and *К*-values was performed according to the recommendations of Landis and Koch [18]. Differences between mean ICC and *К*-values were calculated using the paired Student’s t test. A *p*-value < 0.05 was considered as statistically significant.

## Results

### Intervertebral osteochondrosis

The measurement of the intervertebral disc space showed a good overall inter-observer reliability (ICC: 0.755) and excellent intra-observer reliability (ICC: 0.827) (Tables 1 and 2).

**Table 1:** Inter-observer reliability of intervertebral osteochondrosis (ICC) between different observers (W, K, B, and U) and for different spinal segments (L1/2 to L5/S1)

Segment	Observers						
	W/K	W/B	W/U	K/B	K/U	B/U	Mean
L1/L2	0.698	0.696	0.735	0.751	0.732	0.716	0.721
L2/L3	0.770	0.790	0.686	0.799	0.865	0.787	0.783
L3/L4	0.769	0.791	0.743	0.677	0.858	0.740	0.763
L4/L5	0.817	0.818	0.800	0.731	0.803	0.757	0.788
L5/S1	0.812	0.670	0.708	0.728	0.773	0.676	0.728
Mean	0.773	0.753	0.734	0.737	0.806	0.735	0.757

**Table 2:** Intra-observer reliability of intervertebral osteochondrosis (ICC) for different observers (W, K, B, and U) and for different spinal segments (L1/2 to L5/S1)

Segment	Observers				
	W	K	B	U	Mean
L1/L2	0.842	0.865	0.859	0.766	0.833
L2/L3	0.744	0.881	0.867	0.850	0.836
L3/L4	0.773	0.914	0.800	0.848	0.834
L4/L5	0.817	0.869	0.836	0.837	0.840
L5/S1	0.846	0.848	0.744	0.727	0.791
Mean	0.804	0.875	0.821	0.806	0.827

Inter-observer reliability was significantly lower for the space between the first and second lumbar vertebrae (L1/L2), as compared to that for the space between the fourth and fifth lumbar vertebrae (L4/L5) (*p* = 0.03). The fellowship-trained spinal surgeon (U) showed a significantly better intra-observer reliability than the consultant for orthopedic surgery (K) (p = 0.02) (Tables 1 and 2).

### Vertebral osteosclerosis

Vertebral osteosclerosis showed moderate inter- and intra-observer observer reliabilities (*К*: 0.426, 0.441) (Tables 3 and 4).

**Table 3:** Inter-observer reliability of vertebral osteosclerosis (*К*) between different observers (W, K, B, and U) and for different spinal segments (L1/2 to L5/S1)

Segment	Observers						
	W/K	W/B	W/U	K/B	K/U	B/U	Mean
L1/L2	0,462	0,425	0,423	0,449	0,315	0,357	0,405
L2/L3	0,438	0,462	0,420	0,419	0,479	0,478	0,449
L3/L4	0,438	0,369	0,305	0,391	0,434	0,422	0,393
L4/L5	0,444	0,457	0,396	0,486	0,493	0,450	0,454
L5/S1	0,484	0,480	0,394	0,403	0,391	0,417	0,428
Mean	0,453	0,439	0,388	0,430	0,422	0,425	0,426

**Table 4:** Intra-observer reliability of vertebral osteosclerosis (*К*) for different observers (W, K, B, and U) and for different spinal segments (L1/2 to L5/S1)

Segment	Observers				
	W	K	B	U	Mean
L1/L2	0,469	0,438	0,404	0,400	0,428
L2/L3	0,466	0,418	0,471	0,435	0,448
L3/L4	0,424	0,443	0,398	0,401	0,417
L4/L5	0,486	0,440	0,456	0,451	0,458
L5/S1	0,433	0,454	0,466	0,460	0,453
Mean	0,456	0,439	0,439	0,429	0,441

Inter-observer reliability was significantly lower for the space between the third and fourth lumbar vertebrae (L3/L4) than for the space between the second and third lumbar vertebrae (L2/L3) (*p* = 0.02) and the space between the fourth and fifth vertebra (L4/5) (p = 0.03) (Tables 3 and 4).

### Antero-lateral spondylosis

Antero-lateral spondylosis showed poor inter- and intra-observer reliability (*К*: 0.352, 0.387) (Tables 5 and 6).

**Table 5:** Inter-observer reliability of antero-lateral spondylosis (*К*) between different observers (W, K, B, and U) and for different spinal segments (L1/2 to L5/S1)

Segment	Observers						
	W/K	W/B	W/U	K/B	K/U	B/U	Mean
L1/L2	0,319	0,375	0,311	0,288	0,413	0,382	0,348
L2/L3	0,413	0,348	0,309	0,297	0,274	0,324	0,328
L3/L4	0,340	0,416	0,300	0,458	0,404	0,333	0,375
L4/L5	0,342	0,435	0,351	0,329	0,342	0,334	0,356
L5/S1	0,422	0,399	0,359	0,268	0,318	0,349	0,353
Mean	0,367	0,395	0,326	0,328	0,350	0,344	0,352

**Table 6:** Intra-observer reliability of antero-lateral spondylosis (*К*) for different observers (W, K, B, and U) and for different spinal segments (L1/2 to L5/S1)

Segment	Observers				
	W	K	B	U	Mean
L1/L2	0,406	0,389	0,391	0,339	0,381
L2/L3	0,416	0,393	0,325	0,400	0,384
L3/L4	0,377	0,407	0,373	0,400	0,389
L4/L5	0,425	0,392	0,340	0,393	0,388
L5/S1	0,446	0,397	0,336	0,385	0,391
Mean	0,414	0,396	0,353	0,383	0,387

The consultant for trauma surgery (B) showed significantly lower intra-observer reliability than the orthopedic surgery resident (W) (*p* = 0.05) and the consultant for orthopedic surgery (K) (*p* = 0.03). No significant differences were detected between spinal segments (Tables 5 and 6).

### Posterior spondylosis

Posterior spondylosis showed poor inter- and intra-observer reliabilities (*К*: 0.323, 0.329) (Tables 7 and 8).

**Table 7:** Inter-observer reliability of posterior spondylosis (*К*) between different observers (W, K, B, and U) and for different spinal segments (L1/2 to L5/S1)

Segment	Observers						
	W/K	W/B	W/U	K/B	K/U	B/U	Mean
L1/L2	0,370	0,394	0,296	0,189	0,387	0,268	0,317
L2/L3	0,397	0,352	0,392	0,331	0,383	0,340	0,366
L3/L4	0,291	0,324	0,298	0,324	0,426	0,202	0,311
L4/L5	0,310	0,377	0,286	0,285	0,360	0,344	0,327
L5/S1	0,240	0,310	0,310	0,265	0,265	0,360	0,292
Mean	0,322	0,351	0,316	0,279	0,364	0,303	0,323

**Table 8:** Intra-observer reliability of posterior spondylosis (*К*) for different observers (W, K, B, and U) and for different spinal segments (L1/2 to L5/S1)

Segment	Observers				
	W	K	B	U	Mean
L1/L2	0,382	0,327	0,324	0,419	0,363
L2/L3	0,317	0,397	0,383	0,383	0,370
L3/L4	0,210	0,369	0,268	0,298	0,286
L4/L5	0,337	0,343	0,312	0,318	0,328
L5/S1	0,315	0,316	0,244	0,324	0,300
Mean	0,312	0,350	0,306	0,348	0,329

The inter-observer reliability was significantly lower for the space between the fifth lumbar and the first sacral vertebrae (L5/S1) than for the space between the second and third lumbar vertebrae (L2/L3) (*p* = 0.03). The intra-observer reliability for the space between the fifth lumbar and the first sacral vertebrae (L5/S1) was significantly lower than that for the space between the first and second vertebra (L1/2) (*p* = 0.04). Similarly, the space between the third and fourth vertebra (L3/4) showed significantly lower intra-observer reliability than the space between the second and third vertebra (L2/3) (*p* = 0.02) (Tables 7 and 8).

### Spondyloarthritis

Spondyloarthritis showed poor inter- and intra-observer reliabilities (*К*: 0.275, 0.300) (Tables 9 and 10). The inter-observer reliability was significantly lower for the space between the first and second lumbar vertebra (L1/L2) than that for the spaces between the second and third (L2/L3) (*p* < 0.01) and the fourth and fifth vertebra (L4/5) (*p* = 0.03).

**Table 9:** Inter-observer reliability of spondylarthritis (*К*) between different observers (W, K, B, and U) and for different spinal segments (L1/2 to L5/S1)

Segment	Observers						
	W/K	W/B	W/U	K/B	K/U	B/U	Mean
L1/L2	0,208	0,185	0,209	0,293	0,257	0,233	0,231
L2/L3	0,265	0,227	0,227	0,327	0,327	0,291	0,277
L3/L4	0,281	0,303	0,369	0,227	0,141	0,235	0,259
L4/L5	0,332	0,172	0,357	0,299	0,368	0,376	0,317
L5/S1	0,280	0,343	0,337	0,300	0,260	0,208	0,288
Mean	0,273	0,246	0,300	0,289	0,271	0,269	0,275

**Table 10:** Intra-observer reliability of spondyloarthritis (*К*) for different observers (W, K, B, and U) and for different spinal segments (L1/2 to L5/S1)

Segment	Observers				
	W	K	B	U	Mean
L1/L2	0,322	0,293	0,306	0,302	0,306
L2/L3	0,269	0,237	0,324	0,327	0,289
L3/L4	0,298	0,293	0,292	0,223	0,277
L4/L5	0,322	0,299	0,327	0,363	0,328
L5/S1	0,365	0,320	0,286	0,230	0,300
Mean	0,315	0,288	0,307	0,289	0,300

The orthopedic surgery resident (W) showed a significantly better intra-observer reliability than the consultant for orthopedic surgery (K) (*p* = 0.02) (Tables 9 and 10).

## Discussion

In the present study, the evaluation of intervertebral osteochondrosis by measuring the intervertebral disc spaces of the lumbar spine showed good inter-observer reliability and excellent intra-observer reliability. The classification of vertebral osteosclerosis showed moderate kappa values for both inter- and intra-observer reliability. These data suggest that the 2 parameters, including intervertebral osteochondrosis and vertebral osteosclerosis have sufficient reliability to permit decision making regarding the severity of degenerative occupational diseases of the lumbar spine. In contrast, the classification of lumbar antero-lateral and posterior spondylosis and the evaluation of the spondyloarthritis stage showed poor inter- and intra-observer reliability, which makes these methods unsuitable for daily clinical use.

Based on the DGUV recommendations, intervertebral osteochondrosis should be used as the main parameter for assessing degenerative occupational spinal diseases. Vertebral osteosclerosis, antero-lateral spondylosis, posterior spondylosis, and spondyloarthritis are considered as secondary co-factors that may develop as a result of decreased intervertebral disc spaces, but are not always present. These secondary co-factors should only be used for decision-making in individual cases where the measurement of the intervertebral disc space alone does not lead to a conclusion [10]. Based on our findings, we believe that vertebral osteosclerosis is the only reliable secondary co-factor in addition to intervertebral chondrosis for the assessment of the severity of spinal degeneration.

However, in clinical practice, the measurement and interpretation of intervertebral disc spaces using plain lateral radiographs has certain limitations. First, the radiological projection of vertebral bodies in plain radiographs significantly depends on the position of the central x-ray beam in relation to the spinal segment [19, 20]. Anderson et al. have shown that a lateral tilt of > 10° and an axial rotation of the spine of > 20° can result in significant inter-observer variation when measuring intervertebral disc spaces [19]. Therefore, in patients with lumbar scoliosis, a three-dimensional imaging technique may offer more reliable results relative to plain radiographs. To overcome this problem in the present study, we excluded patients with Cobb angles > 10°.

Similarly, in patients with a history of vertebral fractures, Scheuermann’s disease, or spondylolisthesis, it may be difficult to accurately identify the endplates. Some authors have proposed that in healthy people, the central disc spaces may slightly increase with increasing age, as a result of degenerative microfractures of the endplates that lead to a deformation of the vertebral body [15, 21]. This effect may result in the presence of constant disc spaces over time, even though the patient may have progressive spinal degeneration. Furthermore, there is significant variation between the disc spaces of healthy individuals, which makes it difficult to define reference values for the interpretation of reduced disc spaces in patients with spinal pathologies [15]. Finally, studies have found that intervertebral disc spaces vary significantly during the day depending on the activity pattern of individuals. The delivery of load to the spine during daily activities results in a reduction of water in the nucleus, which reduces the intervertebral space; this effect is reversible during the night [22]. Thus, the measurement of intervertebral chondrosis may produce reduced disc spaces when assessed using radiographs later in the evening, as compared to that on radiographs of the same patient obtained earlier in the morning.

To overcome these problems, Bolm-Audorff et al. developed an algorithm to assess the relative disc space [10, 16]. Therefore, the absolute intervertebral space of a lumbar segments is measured on the lateral x-ray. The values are multiplied with a correction factor to equalize the magnification effects due to radiological projection [15].

The segment with the highest disc space is used as the standard reference. The height of the remaining 4 segments is then calculated as a percentage of the segment with the highest disc space.

In the presented evaluation, inter- and intra-observer reliability of the spaces between the fourth and fifth vertebra were greater than those of other segments. These findings may be consistent with that noted in previous studies, and can be explained by the greater orthogonal geometrical exposure of this segment in relation to the x-ray beam as compared to the others segments [20, 23]. In the present study, the inter- and intra-observer reliability of the intervertebral disc space measurement via plain radiography showed lower ICC values, as compared to those obtained via MRI and ultrasound [24, 25]. Nevertheless, the reliability of the presented method was sufficient for clinical decision making.

However, besides reliability a diagnostic method also requires adequate validity to allow for use in clinical practice. Several trials have shown, that degenerative changes of the lumbar spine do not necessarily correlate with clinical symptoms [26, 27]. Even advanced imaging techniques fail to clearly identify the source of symptoms in patients with low back pain [28]. In addition, recent studies have shown that brain functional changes such as abnormal prefrontal cortex connectivity may also have significant impact on the development and maintenance of chronic back pain [29]. Therefore, future administration of workers compensation for low back pain may require a more comprehensive assessment of co-factors affecting this pathology.

## Conclusions

The measurement of intervertebral osteochondrosis and classification of vertebral osteosclerosis showed an adequate inter- and intra-observer reliability when evaluated using plain radiography. However, the classification of antero-lateral and posterior spondylosis and the spondyloarthritis stage showed insufficient reliability for clinical use. Therefore, these 3 parameters cannot be recommended for the assessment of occupational diseases of the lumbar spine. Thus, we believe that the consensus recommendations of DGUV for the evaluation of occupational diseases of the lumbar spine may require additional revision.

## Data Availability

The datasets analyzed during the current study are available from the corresponding author on reasonable request.

## Abbreviations

DGUV: German Association of Occupational Accident Insurance Funds
HVBG: Federation of Commercial Occupational Insurance Associations
ICC: Intra-class correlation coefficient
*К*: Kappa coefficient
